# Neurological Complications of COVID-19: Unraveling the Pathophysiological Underpinnings and Therapeutic Implications

**DOI:** 10.3390/v16081183

**Published:** 2024-07-24

**Authors:** Ashutosh Vashisht, Vishakha Vashisht, Harmanpreet Singh, Pankaj Ahluwalia, Ashis K. Mondal, Colin Williams, Jaspreet Farmaha, Jana Woodall, Ravindra Kolhe

**Affiliations:** 1 Department of Pathology, Medical College of Georgia, Augusta University, Augusta, GA 30912, USA; avashisht@augusta.edu (A.V.); vvashisht@augusta.edu (V.V.); hsingh1@augusta.edu (H.S.); pahluwalia@augusta.edu (P.A.); amondal@augusta.edu (A.K.M.); jfarmaha@augusta.edu (J.F.); jawoodall@augusta.edu (J.W.); 2 Lincoln Memorial DeBusk College of Osteopathic Medicine, Lincoln Memorial University, Knoxville, TN 37902, USA; colin.williams@lmunet.edu

**Keywords:** NeuroCOVID, SARS-CoV-2, COVID-19, neurological complications, neurological manifestations

## Abstract

Severe acute respiratory syndrome coronavirus 2 (SARS-CoV-2), the causative agent of coronavirus disease (COVID-19), induced a global pandemic with a diverse array of clinical manifestations. While the acute phase of the pandemic may be waning, the intricacies of COVID-19′s impact on neurological health remain a crucial area of investigation. Early recognition of the spectrum of COVID-19 symptoms, ranging from mild fever and cough to life-threatening respiratory distress and multi-organ failure, underscored the significance of neurological complications, including anosmia, seizures, stroke, disorientation, encephalopathy, and paralysis. Notably, patients requiring intensive care unit (ICU) admission due to neurological challenges or due to them exhibiting neurological abnormalities in the ICU have shown increased mortality rates. COVID-19 can lead to a range of neurological complications such as anosmia, stroke, paralysis, cranial nerve deficits, encephalopathy, delirium, meningitis, seizures, etc., in affected patients. This review elucidates the burgeoning landscape of neurological sequelae associated with SARS-CoV-2 infection and explores the underlying neurobiological mechanisms driving these diverse manifestations. A meticulous examination of potential neuroinvasion routes by SARS-CoV-2 underscores the intricate interplay between the virus and the nervous system. Moreover, we dissect the diverse neurological manifestations emphasizing the necessity of a multifaceted approach to understanding the disease’s neurological footprint. In addition to elucidating the pathophysiological underpinnings, this review surveys current therapeutic modalities and delineates prospective avenues for neuro-COVID research. By integrating epidemiological, clinical, and diagnostic parameters, we endeavor to foster a comprehensive analysis of the nexus between COVID-19 and neurological health, thereby laying the groundwork for targeted therapeutic interventions and long-term management strategies.

## 1. Introduction

Severe acute respiratory syndrome coronavirus 2 (SARS-CoV-2) is a novel beta coronavirus that causes a variety of symptoms collectively referred to as coronavirus disease (COVID-19) [1]. Though the devastating COVID-19 pandemic as we experienced it seems to be behind us, much needs to be comprehended about clinical manifestations of COVID-19 infection [2,3]. Early on in the pandemic, it became increasingly clear that clinical manifestations of SARS-CoV-2 infection could range from minor symptoms like fever, cough, and shortness of breath to serious life-threatening respiratory distress and multi-organ failure requiring hospitalization and intensive care unit (ICU) admission. 

Frequently reported among these symptoms were neurological problems such as anosmia, seizures, stroke, disorientation, encephalopathy, and total paralysis [4,5]. Of specific concern is the increased likelihood of mortality observed in COVID-19 patients who require ICU admission secondary to neurological challenges, as well as COVID-19 patients in ICU with neurological abnormalities [5,6]. Additionally, the patients who leave the ICU and recover from their infection are at an increased risk of developing long-term neuropsychiatric and neurocognitive problems such as depression, post-traumatic stress disorder, psychosis, anxiety, and memory impairment [7,8]. The neurological manifestations observed in COVID-19 patients may be due to the direct SARS-CoV-2 mediated injury to the central nervous system or the sequelae of inflammatory cytokines in the storm triggered by COVID-19 infection or secondary to therapeutic interventions. It is also likely that a pre-existing neurological condition may become unmasked or worsen [9]. 

As we transition into the post-pandemic phase of COVID-19, it is crucial to recognize that we identify the neurological complications associated with the virus and gain a comprehensive understanding of its underlying mechanisms. This knowledge will serve as the basis for effectively managing patients clinically and creating specific therapeutic interventions for acute neurological conditions. In this review, we have focused on the neurological manifestations of COVID-19 diseases, potential pathogenic processes, and biomarkers relevant to the diagnosis of neuro-covid. Finally, we have attempted to make a better understanding of this important component of COVID-19 disease, which will hopefully provide some direction in charting the course to more effective management with regard to its nervous system involvement.

## 2. Neuroinvasion of SARS-CoV-2 via Potential Brain Routes 

One of the potential mechanisms of SARS-CoV-2 entry into the nervous system could be direct entry via angiotensin-converting enzyme 2 (ACE2), an established receptor for entry of SARS-CoV-2. ACE2 has been found in human brain vessels, an observation that has since been attributed to its expression in vascular wall pericytes and smooth muscle cells but not in the endothelium lining cerebral capillaries [10]. Although the number of positive neurons was limited, data mining of human brain single-nuclear RNA sequencing (RNA-seq) data revealed expression in the choroid plexus and neocortical neurons (~2% or less) [11]. Also, lower expression of ACE2 was observed in the microglia, endothelial cells, or pericytes in the human brain as compared to the mouse brain. CD147, a surface molecule that is widely expressed in epithelial, neuronal, myeloid, and lymphoid cells, was identified as a SARS-CoV-2 receptor, hence increasing the likelihood of infection in multiple organs including the brain [12]. Cathepsin L activates SARS-CoV-2 Spike in endosomes and can compensate for viral entry into cells that lack TMPRSS2. Neuropilin (NRP1; transmembrane receptors that function as mediators of neuronal guidance and angiogenesis) can also mediate the transport of virus-sized particles from the intranasal region into the brain in a murine study [13]. The findings imply that vascular wall cells in the human brain may express ACE2 at low levels but other non-canonical SARS-CoV-2 receptors such as Cathepsin L and NRP1, which are present in various brain cells, can make them sensitive to the virus. 

Additionally, transmission electron microscopy samples from the frontal lobes of COVID-19 patients revealed the presence of 80–110 nm viral particles with beta coronavirus features [14]. Similarly, coronavirus particles have been found in the neurons of prior viral epidemic victims [15,16]. SARS-CoV-2 may have significant neuroinvasive potential based on the expression of ACE2 in neurons, endothelial cells, and astrocytes [17,18]. Indeed, human brain organoid investigations and in vivo studies in mice overexpressing human ACE2 demonstrated that SARS-CoV-2 can infect neuronal cells [19,20,21]. However, SARS-CoV-2 RNA identification in the brain and cerebrospinal fluid (CSF) yielded inconsistent results [22,23]. As seen in monkeys infected with coronavirus, the endothelium can become infected and serve as an entry site into the brain parenchyma [24]. Investigating how the virus could infiltrate the nervous system could aid in determining the infection’s virulence and the possibility of direct invasion. Several putative entry pathways for SARS-CoV-2 have been hypothesized below based on investigations with previous coronaviruses.

### 2.1. Blood–Brain Barrier

**Figure 1:**
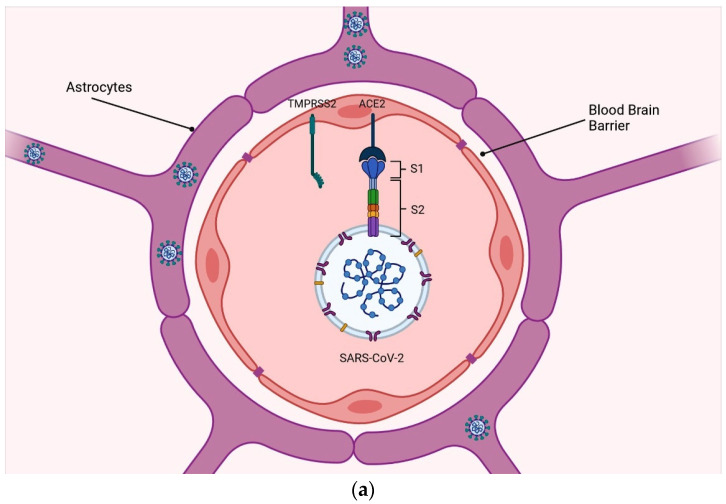

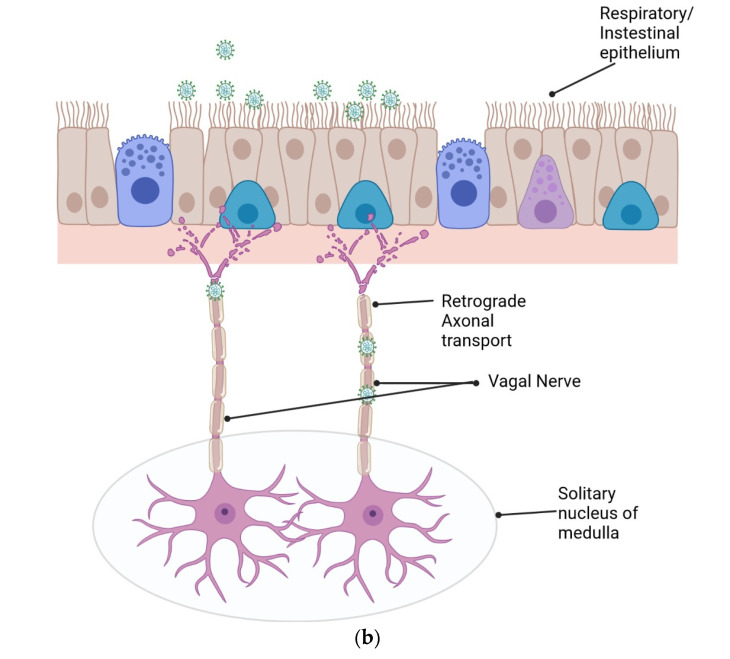
Schematic representation of possible routes through which SARS-CoV-2 could invade the nervous system. (**a**) Blood–brain barrier: the virus may be able to breach this barrier and enter the brain, potentially causing inflammation and other neurological complications; (**b**) Retrograde axonal transport, which refers to the transport of the virus from peripheral nerves back to the brain. Once the virus enters the peripheral nervous system, it may be transported along nerve fibers back to the central nervous system.

Crossing the BBB (blood–brain barrier) is a typical pathway for blood-borne viruses to enter the brain. The virus was found to spread into the circulation in COVID-19 patients, though at varying rates (1% to 41%), and may enter the brain by crossing the BBB [25,26]. Internalization and transport of the virus through the cerebral endothelium, where the expression of SARS-CoV-2 docking proteins is unknown, would be required to cross the intact BBB. Immunoreactivity to ACE2 was found in the brain arteries of a patient who died from repeated ischemic infarctions but the precise cellular location remains unknown [27]. Entry via additional putative SARS-CoV-2 receptors, such as NRP1 and BSG, which are more broadly expressed in the cerebral vasculature, cannot be ruled out. SARS-CoV-2-associated cytokines, such as interleukin (IL)-6, IL-1b, tumor necrosis factor (TNF), and IL-17, damage the BBB, perhaps allowing the virus to enter [28]. SARS-CoV-2 has been suggested to cause endothelial infection and inflammation in peripheral vessels but no direct evidence has been found in brain endothelial cells. Rather, multiple autopsy studies have revealed a lack of florid cerebrovascular inflammation [29,30,31,32]. COVID-19 co-morbidities, such as cardiovascular risk factors or pre-existing neurological disorders, may increase BBB permeability alone or in combination with cytokines. For example, electron imaging detected viral particles in frontal lobe microvessels and neurons in a COVID-19 patient with Parkinson’s disease, implying trans-endothelial entry [33]. SARS-CoV-2 might potentially enter the brain through the hypothalamic median eminence and other circumventricular organs, as well as other brain areas that have a permeable BBB due to fenestrated capillary walls. Although the viral particle is bigger than endothelial fenestrae, preliminary results show that capillaries in the median eminence express ACE2 and TMPRSS, which might facilitate virus passage into the hypothalamus (Figure 1a) [34], which, under its extensive connections, might act as a portal to the whole brain.

### 2.2. Immune Deregulation

Viruses can reach the brain via infected immune cells, which can serve as their reservoir [35]. The vasculature, meninges, and choroid plexus all carry monocytes, neutrophils, and T cells into the brain and these locations might represent entry routes for infected immune cells [36,37]. Until recently, there has been no conclusive proof of the SARS-CoV-2 infection of immune cells. Single-cell RNA seq data revealed viral RNA in macrophages in bronchoalveolar lavage of COVID-19 patients, whereas SARS-CoV-2 nucleocapsid protein (NP) immunoreactivity was detected in CD68+ cells in lymphoid organs [38,39]. It is unclear as to whether this is due to virus replication in macrophages or if it is due to the phagocytic uptake of virus-infected cells or extracellular virions. A notable absence of immune cell infiltration has also been documented in numerous autopsy series [40,41,42].

### 2.3. Retrograde Axonal Transport

For viral transmission along the axon into the perikaryon, retrograde transport machinery can be used (Figure 1b) [43]. Colchicine and vinblastine disrupt microtubules, which impair neural transport and inhibit the reproduction of the murine hepatitis virus (MHV) [44]. Several respiratory viruses, including H5N1, have been found to move from the respiratory system to the brain via the vagus nerve in animal studies [45]. The perikaryon of vagal sensory neurons is located in the solitary nucleus of the medulla, linked to the medulla’s respiratory center and the pons. If the infection reaches the solitary nucleus, the respiratory center may be at risk, leading to respiratory failure. In addition to retrograde transport, SARS-CoV-2, like other coronaviruses, may use the axonal endoplasmic reticulum of infected neurons to spread to the brain [46]. 

### 2.4. Via Gastrointestinal Tract

The virus may be carried from the gastrointestinal tract into the brain because the former is lined by ACE2-expressing cells, contains numerous neurons, and is widely innervated by the vagus nerve [47]. ACE2 cleaves angiotensin (Ang) into Ang (1–7) that binds to Mas receptors and activates anti-inflammatory and anti-fibrosis pathways in the renin–angiotensin system (RAS) in vivo [48,49]. SARS-CoV-2 activates the host immune system by interacting with resident lymphocytes in the intestinal epithelium and lamina propria. The phosphorylation of p38 mitogen kinase and nuclear factor-kB pathways occurs as a result of this activation and the imbalance of RAS-ACE2, resulting in the production of inflammatory factors such as tumor necrosis factor-a, interleukin-1, interleukin-6, and interleukin-8 [50]. As a result, the shedding and death of intestinal epithelial cells is stimulated, increasing the intestinal mucosa’s permeability. On the other hand, the BBB’s tight junction proteins may be damaged, allowing SARS-CoV-2 to enter the brain and cause lesions in the central nervous system in COVID-19 patients. Furthermore, SARS-CoV-2 RNA has been found in rectal swabs and feces samples and it stays detectable for a longer period, even when PCR findings from nasopharyngeal swab testing are negative [51].

## 3. SARS-CoV-2 Variants and Their Neurological Manifestations 

Throughout the COVID-19 pandemic, the genome of SARS-CoV-2 has undergone evolutionary changes, giving rise to new variants that have spread globally. By the end of 2020, three prominent variants had emerged: Alpha (B.1.1.7), Beta (B.1.351), and Gamma (P.1). Subsequently, in May 2021, the Delta variant (B.1.617.2) emerged, followed by the Omicron variant in October (B.1.1.529) [52]. These variants differ in their ability to spread, evade immunity, and affect the severity of COVID-19. Importantly, various studies have reported differences in the incidence of neurological disorders associated with these different variants. For instance, initial research indicated that 8.4% of patients likely infected with the original Wuhan-Hu-1 strain developed neurological issues. This proportion decreased to 5.0% during the prevalence of the Alpha variant and further declined to 3.3% during the Delta variant surge [53]. Similarly, animal studies, such as the one conducted with hamsters, have shown that the Wuhan strain induces anosmia more frequently than the Gamma variant, with no instances of anosmia observed in hamsters infected with the Delta or Omicron variants [54].

In a separate investigation conducted by Taquet et al., an extensive cohort analysis scrutinized neurological and psychiatric conditions among patients afflicted with COVID-19 before the onset of the Alpha variant, alongside those infected with Delta, or Omicron variants. The results notably demonstrated an elevated susceptibility among Delta-infected individuals to develop ischemic stroke, epilepsy, seizures, and cognitive impairments [55]. Distinct clinical manifestations correlate with differential susceptibility of nervous system cells. Recently, Proust et al. published a study comparing the infectivity and replication efficiency of the Wuhan strain, Alpha, Beta, Delta, Eta, and Omicron (BA.1) within blood–brain barrier (BBB) cells [56]. Their findings revealed varying susceptibilities, with Alpha, Beta, and Omicron showing productive infection in astrocytes; Wuhan, Beta, and Omicron in epithelial cells; Omicron exclusively in pericytes; and Wuhan, Delta, and Omicron variants in microglial cells.

Furthermore, the delta variant (B.1.617.2) of SARS-CoV-2 exhibited the highest risk among COVID-19 variants for neuropsychiatric complications and mortality. Specifically, there was a notable increase in cognitive deficits, epilepsy, and ischemic stroke incidences compounded by an elevated mortality risk [57]. During the periods dominated by the alpha variant (B.1.1.7) and the omicron variant (B.1.1.529), the neurological and psychiatric complication profiles closely mirrored those observed during the delta variant pandemic phase. There were no significant differences in the risks of cognitive deficits, epilepsy, ischemic stroke, psychotic disorders, or mood disorders among these variants. Despite similar rates of neurological complications, the omicron variant showed a reduced composite risk of hospitalization and death, likely due to a less severe course of SARS-CoV-2 infection [55,57].

Additionally, COVID-19 patients have had a wide range of neurological symptoms (Table 1). In mild cases, hyposmia and hypogeusia are seen, while severe cases of COVID-19 show agitation, diffuse corticospinal tract symptoms, and myoclonus [58,59,60]. Female patients are more affected than male ones but the severity is much less than in males [61,62]. Other data indicated that neurological symptoms such as a loss of smell and taste occurred in 19% of individuals and some reported ataxia, seizures, vision impairment, and other symptoms as low as 5% [63,64]. Because the symptoms are linked to a milder clinical course or cases without respiratory symptoms, the source of the disparity might be related to anamnesis techniques and a variation in the degree of illness severity [65]. An objective investigation employing a scent-identification (40 odorants) test indicated that 98 percent of individuals with COVID-19 had impaired smell function. In total, 58 percent of them were anosmic or severely microsmic [66]. In comparison to the real number of COVID-19 cases, the number of reported participants in such trials is still rather modest and additional data are needed. Nonetheless, anosmia and ageusia have been added to the list of symptoms and risk factors linked with COVID-19, according to the most recent COVID-19 clinical treatment guidelines [67]. Symptoms of muscle involvement, such as fatigue and limb pain or soreness are also linked. Dizziness and headache, decreased awareness, seizures, psychiatric symptoms, diffuse corticospinal tract indications, ataxia, and acute cerebrovascular events, such as ischemic stroke and cerebral hemorrhage, have all been reported as CNS involvement [68,69]. 

Also, there is evidence that viral proteins may survive in people for months and can affect various systems even if they do not infect cells. The SARS-CoV-2 N protein was identified in intestinal enterocytes in 5 of 14 people after an average of 4 months (range of 2.8 to 5.7 months) post-COVID-19 diagnosis, which may imply its participation in extended COVID [70]. Various neurological disorders have been reported to occur weeks, months, or even years after acute viral infection in survivors of previous pandemics. Between three and four weeks after SARS-CoV and MERS-CoV infection, neurological problems such as limb weakness, hyporeflexia, paresthesia, and hypesthesia were described [71,72]. Indeed, mice who survived HCoV-induced acute encephalitis had lower locomotor activity, a smaller hippocampus, and neuronal loss in the CA1 and CA3 layers, all of which might lead to learning and memory deficits [73]. Immune responses to viral infection have been linked to the onset of neurological autoimmune disorders such as multiple sclerosis (MS) [74]. The causes, which include persistent inflammation caused by prolonged IFN production, have recently been explored [75]. Furthermore, HCoV-OC43 was found in the brain tissues of 48 percent of MS patients [76]. The demyelinating effect of HCoV-229E in MS patients has been confirmed by investigating mouse hepatitis virus (MHV)-infected mice [77]. More research on how the immune system responds to viral infection and how this relates to the prevention of virus-related autoimmune disorders is essential. The involvement of the gastrointestinal tract in SARS-CoV-2 infection poses an additional hazard to the neurological system, since it may enhance the induction and transit of abnormal proteins such as α-synuclein, which is linked to neurodegenerative disorders [78]. Inflammation-induced bacterial leakage from the gastrointestinal system may transport bacteria and metabolites into the brain parenchyma, causing CNS disease [79]. 

At the molecular level, severe COVID-19 infection can induce epigenetic changes in the frontal cortex that resemble those of natural aging, such as DNA methylation and histone modifications [80]. COVID-19 infection can also trigger synapse elimination in microglia cells and upregulate cytokines. The single cell profiling of SAR-SoV-2-infected patients has observed upregulation of synapse engulfment and migration along with other immune-related genes such as TNF-α and IL-6. The disruption of normal brain circuitry can promote a cognitive decline in patients after recovery from COVID and stress-related responses such as mood alterations [81]. Furthermore, SARS-CoV-2 can affect normal mitochondrial physiology, which is essential for maintaining cellular energy reserve and homeostasis. Mitochondrial dysfunctionality contributes to the buildup of oxidative stress, inflammation, impaired synaptic plasticity, and inflammation. Mitochondrial malfunction is linked to several degenerative disorders, including Alzheimer’s, Parkinson’s, and Huntington’s. Furthermore, aging is also associated with mitochondrial anomalies and the dysregulation of cellular bioenergetics [82]. Furthermore, mitochondria are hijacked by SARS-CoV-2 of the immune cells and utilize these as replicative niches [83]. Additional factors such as diabetes, cardiovascular diseases, and age-associated vulnerabilities further complicate clinical etiological diseases [84,85].

Also, cytokines have been shown to play a critical role in mood disorders. For example, the upregulation of TNF-α and IL-6, two major cytokines in COVID-19, directly affects brain physiology and may negatively promote stress-related responses such as mood alterations [86].

**Table 1:** Neurological symptoms of COVID-19 according to the patients’ pathophysiological background.

S. No.	Patient Size	Sample Type	Type of Study	Study Design	Neurological Manifestation	Altered Markers	Potential Associated Variant	Assay Procedure	Ref.
1	11 COVID-19 patients	Serum and CSF	Clinical	Retrospective	Myoclonus, oculomotor disturbance, delirium, dystonia, and epileptic seizures	Neurofilament light chain (NfL) levels in CSF were increased in all tested patients. one patient showed Yo antibodies in serum and CSF and two patients myelin antibodies in serum. One patient had high-level serum IgG NMDA receptor antibodies	SARS-CoV-2 Beta	Indirect immunofluorescence assay	[87]
2	18 COVID-19 subjects, 14 healthy controls, and 68 non-COVID-19 neurological disease controls	CSF	Clinical	Retrospective	Stroke, encephalopathy, and headache	Anti-SARS-CoV2 antibodies were found in 77% of COVID-19 subjects’ CSF. Increase in pro-inflammatory cytokines (IL-6, TNFα, and IL-12p70) and IL-10 in the CSF of COVID-19 patients. CSF-hsCRP was present exclusively in COVID-19 cases	SARS-CoV-2 Beta	RT-PCR and ELISA	[88]
3	35 COVID-19 patients	Serum/CSF	Clinical	Retrospective	Encephalitis and stroke	CSF tumor necrosis factor-alpha (TNFα) and IL6 levels were higher in patients presenting pronounced neuroimaging alterations compared to those who did not	SARS-CoV-2 Beta	Cytokines analysis using magnetic beads, ELISA, and LC-MS/MS	[89]
4	844 COVID-19 patients	Blood	Clinical	Retrospective	Stroke and intracranial hemorrhage	Anticardiolipin antibodies were observed in all the patients	SARS-CoV-2 Beta	Biochemical analysis	[90]
5	12 COVID-19 patients and 19 healthy controls	Plasma	Clinical	Longitudinal analysis/Prospective	Difficulty with memory or concentration, increased anxiety, and depression	IL-1β was significantly increased in COVID patients, both brain-derived neurotrophic factor and cortisol were significantly elevated in COVID patients	SARS-CoV-2 Epsilon, Alpha, Delta, and Omicron	Genotyping analysis, cytokine analysis, and ELISA	[91]
6	400 COVID-19 patients	Plasma	Clinical/Autopsy	Retrospective	ischemic stroke, dementia, epilepsy, and hemorrhagic stroke	LDH, ferritin, hsTnI, and IL-6 were found to be higher in deceased patients. A concentration of hsTnI > 64 ng/L appeared to constitute a strong predictor of an unfavorable prognosis	SARS-CoV-2 Epsilon, Alpha, and Delta	RT-PCR, MR, CT, Doppler US imaging, and biochemical analysis	[92]
7	72 COVID-19 Patients	Blood	Clinical	Prospective	Fatigue, anxiety, and depression	Elevated levels of TNF-α and IL-1β were associated with decreased episodic memory, working memory, and inhibitory control	SARS-CoV-2 Beta and Delta	Biochemical analysis	[93]

## 4. Therapeutic Strategies

At this time, there is no specific proven therapeutic regimen for neurological complications associated with COVID-19, regardless of the underlying mechanism. While acute stroke therapies and seizures have not been specifically tested in the COVID-19 population, there are no data to suggest that the risk–benefit ratio of these interventions differs for patients with COVID-19. Therefore, the management of ischemic and hemorrhagic stroke in COVID-19 should follow the same standards of care as for patients without COVID-19 (Table 2). This includes a timely evaluation of candidacy for acute medical and interventional stroke therapies, such as thrombolysis or thrombectomy in acute ischemic stroke [94,95]. For encephalopathy, reported options include monotherapy or combinations of corticosteroids (for example, methylprednisolone 1 g daily for 5–10 days), intravenous immunoglobulin, plasma exchange, and rituximab [96]. Furthermore, in GBS, it should only be considered for COVID-19 if respiratory insufficiency seems disproportionate to pulmonary findings. Also, with other causes of GBS, both intravenous immunoglobulin and plasma exchange are reported therapies that could be explored in COVID-19 [97].

The autonomic nervous system’s immunomodulatory actions may be useful in preventing the hyper-inflammatory state that leads to severe COVID-19 symptoms. Maintaining a small but efficient immune response may allow infections to be eliminated while minimizing tissue damage, including CNS involvement. Some neurons, such as those in the olfactory bulb of mice, can withstand coronavirus infection. Differential gene expression in those neurons may yield useful information that may be used to develop neuroprotection methods for acute neurotropic virus infection. IL-10 therapy in MHV-infected mice has been shown to cause astrocytes to produce glial scars that restrict demyelination regions [98]. Remyelination may occur after myelin breakdown, as evidenced by overexpression of genes involved in oligodendrocyte maturation, such as Oncostatin M, in mice infected with MHV [99]. It is still unclear if the degree of remyelination has an impact on the disease’s prognosis. The inhibition of CD147, one of the SARS-CoV-2 receptors, has been shown in mouse models of ischemic stroke to offer further benefits in preserving oligodendrocytes and white matter [100]. Antibodies against CD147, on the other hand, permeabilize BBB by binding to CD147 in brain endothelial cells, therefore increasing brain inflammation. This double-edged sword impact might thus be beneficial or harmful. Previous research has revealed the possibility of neurological problems associated with immunization. Following vaccination for influenza, polio, rabies, meningococcus, measles, mumps, and tetanus, acute inflammatory demyelinating polyneuropathy (AIDP) is the most frequent neurologic condition, as documented in case reports [101]. Despite these limitations, the currently available data show extremely modest rates of neurologic sequelae following immunization, especially when compared to the severity of infection-related morbidity and death in populations of individuals with neurological disease. 

## 5. Future Perspective

The field of neuro-COVID research is still in its early stages and much is yet to be learned about the long-term effects of COVID-19 on the brain and nervous system. However, several potential future perspectives could emerge from the ongoing research.

1. Understanding the mechanisms of COVID-19’s impact on the brain: More studies should be performed to enhance the understanding of the mechanisms by which the SARS-CoV-2 impacts the neuro system. As more time passes since the beginning of the pandemic, researchers will be able to study the long-term effects of COVID-19 on the brain and nervous system. This could provide important insights into the potential long-term consequences of COVID-19, such as increased risk of dementia or other cognitive impairments;
2. Development of new diagnostic tools: Neuro-COVID research could lead to the development of new diagnostic tools to detect neurological complications of COVID-19. For example, the use of MRI and other imaging techniques to detect brain changes in COVID-19 patients should be explored;
3. Treatment and management of neurological symptoms: Finally, research into the neurological effects of COVID-19 could lead to new treatments and management strategies for patients experiencing these symptoms. A study published in the Journal of Neuroimmunology in 2021 found that a combination of two monoclonal antibodies, casirivimab and imdevimab, improved neurological symptoms in hospitalized COVID-19 patients [102]. Another study published in the Journal of Clinical Investigation in 2021 found that a monoclonal antibody called CT-P59 was effective in reducing COVID-19 symptoms, including neurological symptoms, in non-hospitalized patients [103]. Also, there is a growing interest in non-pharmacological interventions for the treatment of NeuroCOVID. These interventions may include therapies such as physical rehabilitation, cognitive–behavioral therapy, and neuropsychological assessments. A study published in the Journal of Neurologic Physical Therapy in 2021 found that physical therapy was effective in improving balance and gait in COVID-19 patients with neurological complications [104].

Overall, the future perspectives on the therapeutics of NeuroCOVID are promising, with a range of potential treatments and approaches being explored. As our understanding of the neurological complications of COVID-19 continues to evolve, new therapies and interventions will likely emerge to help those affected by this condition.

## Figures and Tables

**Table 2:** Management of neurological complications associated with COVID-19: therapeutic strategies and considerations.

Aspect of Management	Treatment Details
Acute Stroke Therapies	Acute stroke therapies (thrombolysis and thrombectomy) not specifically tested in COVID-19 population; standard care applies as for non-COVID-19 patients. Evaluation for candidacy should be timely.
Seizure Management	No specific data on seizure management in COVID-19 patients; general risk–benefit ratio applies.
Encephalopathy Treatment Options	Reported options include monotherapy or combinations of corticosteroids (e.g., methylprednisolone 1 g daily for 5–10 days), intravenous immunoglobulin, plasma exchange, and rituximab.
Ischemic and Hemorrhagic Stroke Management	Follow same standards of care as non-COVID-19 patients, including timely evaluation for acute medical and interventional stroke therapies.
Guillain-Barré Syndrome (GBS)	Consider if respiratory insufficiency is disproportionate to pulmonary findings in COVID-19 patients. Intravenous immunoglobulin and plasma exchange are reported therapies.
Autonomic Nervous System	Immunomodulatory actions may help prevent the hyper-inflammatory state, leading to severe COVID-19 symptoms. Maintaining an efficient immune response may minimize tissue damage, including CNS involvement.
IL-10 Therapy	No study comprising human samples so far but in MHV-infected mice, IL-10 therapy causes astrocytes to produce glial scars that restrict demyelination regions.
CD147 Inhibition	In mouse models of ischemic stroke, the inhibition of CD147 (a SARS-CoV-2 receptor) helps preserve oligodendrocytes and white matter. However, antibodies against CD147 can increase brain inflammation, presenting a double-edged sword effect.
